# Combination of a Novel Genetic Variant in *CFB* Gene and a Pathogenic Variant in *COL4A5* Gene in a Sibling Renal Disease: A Case Report

**DOI:** 10.3389/fgene.2021.690952

**Published:** 2021-07-19

**Authors:** Feng-mei Wang, Yan Yang, Xiao-liang Zhang, Yan-li Wang, Yan Tu, Bi-Cheng Liu, Bin Wang

**Affiliations:** Institute of Nephrology, Zhongda Hospital, Southeast University School of Medicine, Nanjing, China

**Keywords:** complement factor B, *COL4A5*, variant, complement alternative pathway, renal disease, case report

## Abstract

*Complement factor B* (*CFB*) variants have been described to play a causative role in auto-immune associated C3 glomerulopathy (C3G) and/or atypical hemolytic uremic syndrome (aHUS) by affecting the dysregulations of alternative pathway activation. However, *CFB* variant concomitant with *COL4A5* variant is scarce. Here, we depict two intriguing cases with concurrent novel heterozygosity for *CFB* c.2054_2057del (p.Ser687Profs^∗^16) variant and a previous reported *COL4A5* c.2999G > T (p.Gly1000Val) variant in a pair of siblings. The clinical feature of either paternal *CFB* variant or maternal *COL4A5* variant is just mild microscopic hematuria. Interestingly, their two children with paternal *CFB* c.2054_2057del (p.Ser687Profs^∗^16) variant and maternal *COL4A5* c.2999G > T (p.Gly1000Val) variant presented with massive proteinuria, hematuria, and progressive renal failure with poor treatment response. Moreover, complement pathway activation in renal tissue further supports and strengthens the pathogenic role of *CFB* variant in the development of renal injury in the presence of *COL4A5* variant. In conclusion, the rare sibling cases highlight that the extension of genetic analyses in the proband is helpful for the diagnosis and understanding of some family cluster renal diseases.

## Introduction

Factor B (FB) is the initial factor of complement alternative pathway (AP) activation. Generally, it is cleaved by complement factor D into two fragments, Ba (residues 1–234) and Bb (residues 235–739), respectively. Bb fragment as a serine protease (SP) consists of a C-terminal serine protease domain and an N-terminal von Willebrand factor type A (vWA) domain. It combines with the cleavage product of complement protein C3 (C3b) to form C3 or C5 convertases and plays an essential role in the amplification of complement activation (Janssen et al., 2009). *CFB* gene variants are associated with C3 glomerulopathy (C3G), atypical hemolytic uremic syndrome (aHUS), and recurrent infections by affecting the dysregulations of AP activation (Imamura et al., 2015; Sethi et al., 2015; Alfakeeh et al., 2017; Zhang et al., 2020).

*COL4A5* is closely associated with Alport syndrome (AS) (Kaneko et al., 2010; Rheault et al., 2020). However, *CFB* variant concomitant with *COL4A5* variant is scarce. Herein, we present a pair of siblings with the c.2054_2057del (p.Ser687Profs^∗^16) novel genetic variant in the *CFB* gene and the c.2999G > T (p.Gly1000Val) reported pathogenic variant in the *COL4A5* gene. To the best of our knowledge, this is the first case report that demonstrates the involvement of *CFB* variant in the etiology of glomerular nephropathy concomitant with a *COL4A5* pathogenic variant.

## Case Description

An 18-year-old boy referred to our hospital on January 14, 2020, complained about proteinuria and hematuria for 8 months. Physical examination was unremarkable with neither hearing loss nor ocular abnormalities.

Biochemistry analysis revealed serum total protein 62.5 g/L, albumin, 40.4 g/L and creatinine, 85 μmol/L (57–111 μmol/L). By urinalysis, protein excretion was 2.3 g/day and RBCs were 24/HPF. Immunological evaluation was as follows: C3, 1.18 g/L; C4, 0.254 g/L (normal range, 0.9–1.8 g/L; 0.1–0.4 g/L, respectively). Serology for antinuclear, anti-double-stranded DNA, anti-streptolysin O, antiphospholipid, anti-glomerular basement membrane (GBM), anti-neutrophilic cytoplasmic antibodies (ANCA), anti-PLA_2_R antibodies, and serum immunofixation electrophoresis were all negative. There was no serological evidence of hepatitis B and C or HIV infections.

**FIGURE 1:**
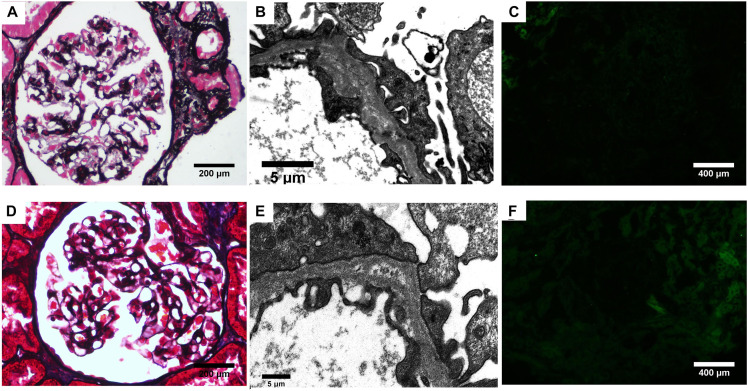
Renal biopsy findings. **(A–C)** Biopsy from the proband. **(A)** Mild mesangial cells and matrix proliferation is seen (PASM). **(B)** Lamina densa lamellation and splitting is seen along the GBM on electron microscopy. **(C)** Complete absence of α5 staining in the glomerular and tubular basement membranes. **(D–F)** Biopsy from the elder sister. **(D)** Segmental mesangial cells and matrix proliferation is seen (PASM). **(E)** Electron microscopy revealed splitting GBM. **(F)** The α5 staining in the glomerular and tubular basement membranes was absent.

Then, kidney biopsy was performed. Sixteen glomeruli were sampled for light microscopy with two spheroidal sclerosis. Light microscopy showed glomerular mesangial mild hyperplasia, mild tubulointerstitial lesions (Figure 1A). Immunofluorescence revealed all negative for IgA, IgG, IgM, C3, C1q, and Fibrin. Electron microscopy demonstrated mesangial electron-dense deposits, podocyte foot process effacement, and segmental GBM with splitting appearance (Figure 1B). The GBM changes raised concern for Alport syndrome (AS), and additional immunofluorescence was performed for type IV collagen α1, α3, and α5 chains. However, α5 was completely absent in both glomeruli and tubules (Figure 1C).

Regarding the family history, his elder sister was initially presented with hematuria and proteinuria in her 9th year of age. Light microscopy of kidney biopsy showed segmental mesangial proliferative glomerulonephritis (Figure 1D). Electron microscopy finding revealed splitting of GBM (Figure 1E). Immunofluorescence was all negative including α5 chain (Figure 1F). However, she had discontinued periodic examination for 18 years. In September 2020, she was admitted to the outpatient department of our hospital with proteinuria and hematuria. Urine analysis detected 82 dysmorphic RBCs/HPF, 2.1 g/d proteinuria. Biochemistry analysis showed serum total protein 64.3 g/L, albumin, 39.2 g/L, and serum creatinine, 123 μmol/L. Furthermore, their father, mother, and paternal aunt all had microscopic dysmorphic hematuria with normal renal function, and without proteinuria.

**FIGURE 2:**
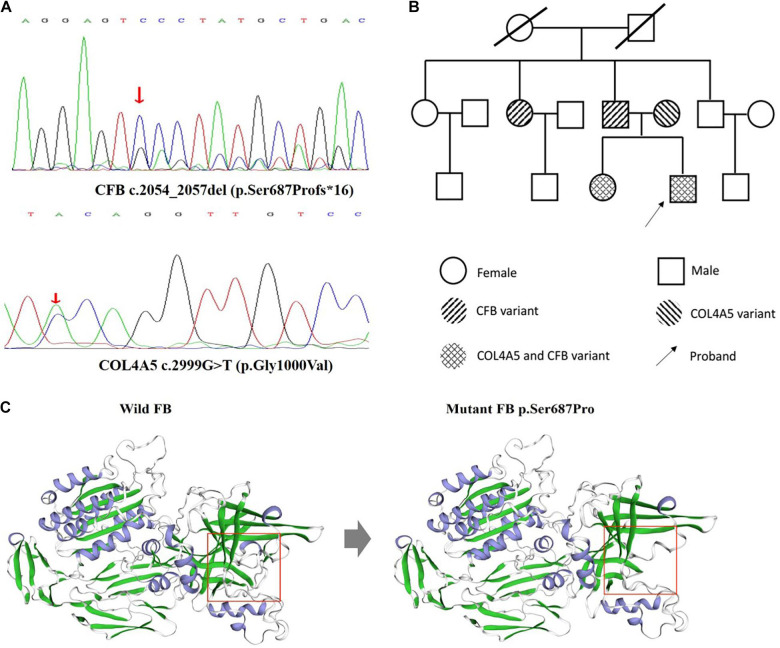
DNA analysis of the patient and family members and structural evaluation of mutant FB p.Ser687Pro. **(A)** Direct sequencing of patient genomic DNA shows the c.2054_2057del (p.Ser687Profs*16) novel genetic variant in exon 16 of the *CFB* gene, c.2999G > T (p.Gly1000Val) in exon 34 of the *COL4A5* gene. **(B)** The pedigrees of the family with digenic inheritance. **(C)** Visualization of the mutant FB. The figure was prepared using swiss-model. Pr, proteinuria; He, hematuria. Red arrow means the position of mutation. Red square means the impact of the frameshift mutation on splice site changes and protein features.

Considering the unusual pathological features and family medical history, next-generation sequencing was performed for the proband. The result demonstrated *CFB* c.2054_2057del (p.Ser687Profs^∗^16) variant and X-linked *COL4A5* c.2999G > T (p.Gly1000Val) variant (Figure 2A). Sanger sequencing analysis of the family members revealed *CFB* c.2054_2057del (p.Ser687Profs^∗^16) variant in the patient’s father and paternal aunt, X-linked *COL4A5* c.2999G > T (p.Gly1000Val) variant in his mother. And his elder sister has the same genetic variants as the proband (Figure 2B).

*In silico* analysis indicated the pathogenic nature of the c.2054_2057del (p.Ser687Profs^∗^16) novel genetic variant in CFB gene. The frameshift mutation has been predicted by rare exome variant ensemble learner as “likely pathogenic,” which has an impact on splice site changes and protein features might be affected (Figure 2C). Additionally, X-linked *COL4A5* c.2999G > T (p.Gly1000Val) variant has been reported in a family with hematuria without a renal biopsy (Kaneko et al., 2010).

**FIGURE 3:**
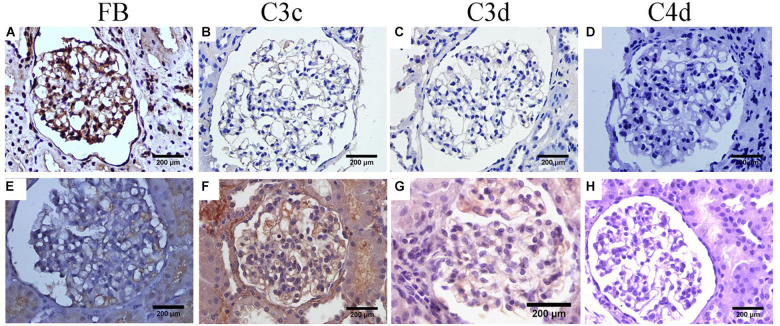
Immunohistochemical staining of complement components. **(A–D)** For the proband. **(E–H)** For his elder sister. **(A,E)** FB staining within glomerular capillary and segmental mesangial for the proband and his elder sister, respectively. **(B,F)** C3c staining mainly along glomerular capillary for the proband and his elder sister, respectively. **(C,G)** Weak glomerular staining with C3d along glomerular capillary for the proband and his elder sister, respectively. **(D,H)** Absent glomerular C4d staining for the proband and his elder sister, respectively.

Furthermore, we performed immunohistochemical staining for Factor B (FB), C3c, C3d, and C4d in renal tissue to determine the complement activation pathway. The result demonstrated that FB, C3c, and C3d were all positive in the proband and his elder sister, C4d, was negative in glomeruli (Figure 3). Furthermore, the plasma FB levels in the proband and his sister were 0.168 g/L and 0.3 g/L (normal range: 0.19–0.5 g/L), respectively. At this time, the proband and his sister were diagnosed as CFB c.2054_2057del (p.Ser687Profs^∗^16) variant associated glomerular nephropathy combined with X-linked Alport Syndrome.

The patient was treated with initial prednisone (35 mg/d, gradually reducing the dose taken by 5 mg every 8 weeks), Benazepril (10 mg/d), and Hydroxychloroquine sulfate (0.2 g, bid) for 6 months. Now, oral administration of 25 mg prednisone continued. At present, his proteinuria persisted and increased to 3.0 g/d, and serum creatinine reached 130 μmol/L with renal insufficiency.

## Discussion

Herein, we describe two sibling cases of *CFB* variant coexistence with Alport syndrome. In the family, we found *CFB* c.2054_2057del (p.Ser687Profs^∗^16) variant from father and *COL4A5* c.2999G > T (p.Gly1000Val) variant from mother, while the parents and their paternal aunt only manifest with hematuria without proteinuria with normal renal function. But when *CFB* c.2054_2057del (p.Ser687Profs^∗^16) variant inherited from father came across *COL4A5* c.2999G > T (p.Gly1000Val) variant inherited from mother, the clinical feature was more severe, accompanying with progressive renal failure, which suggested *CFB* c.2054_2057del (p.Ser687Profs^∗^16) variant might be a pathogenic role in the etiology of glomerular injury combined with Alport syndrome.

Alport syndrome is a rare genetic and progressive glomerular disease, caused by the variants in the *COL4A3*, *COL4A4*, or *COL4A5* genes (Wei et al., 2006; Heidet and Gubler, 2009; Savige et al., 2013). In our study, X-linked *COL4A5* c.2999G > T (p.Gly1000Val) variant in exon 34, has been reported in a family with hematuria without a renal biopsy (Kaneko et al., 2010).

Different types of genetic variants in the *COL4A5* gene determined different distinct phenotypes in X-linked AS. Specifically, the X-Linked AS has continuum phenotype but can be didactically classified into three forms: severe form, moderate-severe form, and mild-moderate form. The mild-moderate form is usually caused by glycine-XY variants not involving the NC1-domain (Gross et al., 2002; Slajpah et al., 2007). In our study, their mother presented with simple hematuria with neither hearing loss nor ocular abnormalities, so the mild-moderate form of X-linked AS was classified. Additionally, X-linked AS also existed in the two siblings according to hematuria, splitting of the lamina densa in GBM, and X-linked *COL4A5* c.2999G > T (p.Gly1000Val) variant.

**FIGURE 4:**
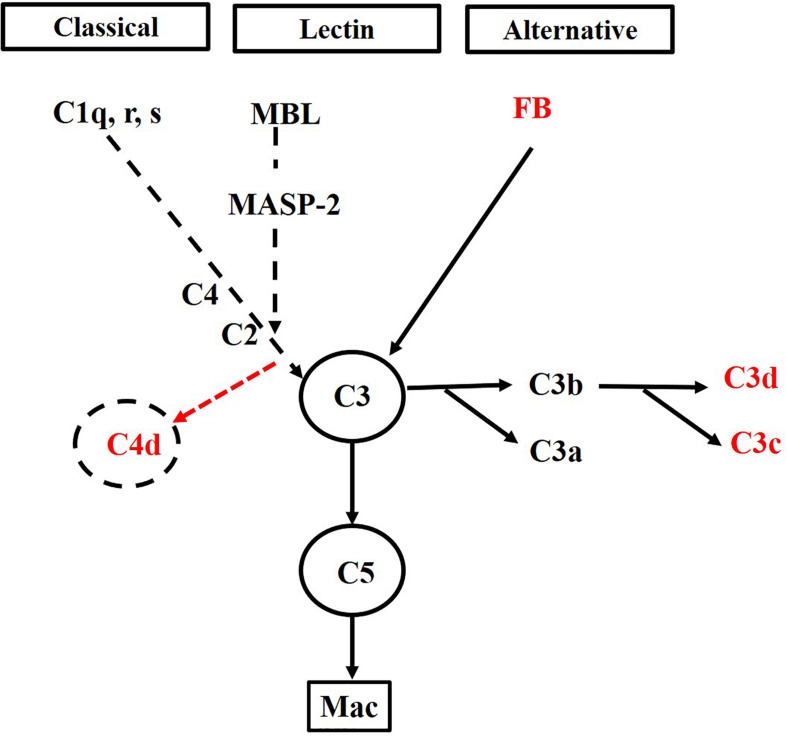
The schematic diagram of complement activation pathway in our patients. Three pathways of complement activation exist: the classical, mannose-binding lectin (MBL), and alternative pathways. Activation of the classical pathway and the lectin pathway can cleave and activate C4 and C2 to form C4bC2a, which subsequently results in the formation of C4d. Activation of any of the three pathways leads to activation of C3, which generates C3a and C3b. Furthermore, C3b can generate C3c and C3d. The absence of C4 deposition in our patients suggests that both the classical and lectin complement pathways are not involved. On the contrary, the presence of FB, C3c, and C3d deposits demonstrates that the alternative complement pathway is activated in our patients.

Factor B plays an essential role in AP activation and protecting the host from opportunistic infections (Slade et al., 2013). In our report, the father transmitted a novel *CFB* c.2054_2057del (p.Ser687Profs^∗^16) variant. And this genetic variant in the father and paternal aunt presented with only mild hematuria. However, when mild phenotype *COL4A5* c.2999G > T (p.Gly1000Val) variant was superposed with a novel *CFB* c.2054_2057del (p.Ser687Profs^∗^16) variant in their children, the pathological injury and clinical manifestation changed vastly, which implies that this genetic variant in CFB gene plays a pathogenic role in the process. Consequently, we conducted complement activation pathway to explore the etiology and pathogenesis of mutant FB. For the proband and his sister, the depositions of FB, C3c, and C3d in the glomeruli were all positive. However, C4d, which was negative, was positive when MBL and/or classical complement pathways was activated (schematic diagram of the complement activation pathway in our patients was shown in Figure 4). These data suggested alternative complement activation participated in the pathogenesis of renal injury and might be a dominant driver in presence of mild phenotype AS.

Given the involvement of AP, it is likely that *CFB* c.2054_2057del (p. Ser687Profs^∗^16) variant plays a pathogenic function through a structure-function relationship. FB is a mosaic protein consisting of three different types of protein modules: three short consensus repeats, one vWA domain, and one SP domain. Activity of the SP catalytic site is strictly regulated by an assembly process that culminates in a C3bBb complex, the active C3 convertase (Janssen et al., 2009). The proband shows the c.2054_2057del (p. Ser687Profs^∗^16) frameshift genetic variant with a premature interruption of the FB coded by this gene. Such a pathogenic variant is supposed to be pathogenic due to a loss of function. The c.2054_2057del (p. Ser687Profs^∗^16) variant in *CFB* gene is just located in SP domain, which might form a dysregulated C3 convertase that causes dysregulation of the alternative pathway activation. With respect to the underlying mechanism of the variant pathogenicity, it is necessary to clarify it by performing the complement assays and functional experiments. We hope that we could furthermore clarify it at the variant level to gain insight into the disease phenotype in the future.

As well known, *CFB* variants have been causally linked to C3G and aHUS. Several studies have clarified that either aHUS or C3G is caused by dysregulation of the alternative pathway of complement. The two diseases share phenotypic similarities and underlying genetic commonalities. Bu et al. (2016) have demonstrated that compared with aHUS, patients with C3G had a higher frequency of rare and novel variants in C3 convertase (*C3* and *CFB*) and complement regulator (*CFH*, *CFI*, *CFHR5*, and *CD46*) genes. In contrast, patients with aHUS had an increase in rare and novel variants only in complement regulator genes, especially *CFH*, a distinction consistent with differing sites of complement dysregulation in these two diseases (Bu et al., 2016). However, a recent study by Zhang et al. (2020) described the functional characterization of a novel *CFB* (c.1101 C > A, p.Ser367Arg) variant in the vWA domain of FB that they identified in two unrelated aHUS pedigrees. Interestingly, the same amino acid variation resulting from a different nucleotide change (*CFB* c.1099A > C; p.Ser367Arg) has been reported in a Japanese patient who had C3G and not aHUS. As compared to the cases described by Zhang et al. (2020), one important difference is that the C3G patient carries two other ultra-rare genetic variants–one in *CFI* (c.603A > C, p.Arg201Ser) and the other in *C3* (c.2746G > A, p.Val916Ile) (Imamura et al., 2015). The above two cases described also call into question the pathogenesis of aHUS and C3G, and suggest that the final disease phenotype is based on a host of modifying genetic and perhaps environmental triggers. According to the previous studies, most aHUS or C3GN- associated FB variants cluster in the vWA domain of Bb, often close to the Mg^2+^ adhesion site (MIDAS). Functional studies have revealed that FB variants affecting vWA and MIDAS domains result in faster association and stronger binding to C3b, and/or to a more stable C3 convertase that is resistant to accelerated decay by the complement regulators, by FH (Noris and Remuzzi, 2020). The result is increased enzyme activity, with the formation of massive amounts of C3 activation products, and a contribution to the pathogenesis of aHUS or C3G. As for our patients, the novel c.2054_2057del (p.Ser687Profs^∗^16) frameshift genetic variant causes a premature interruption of the FB coded by this gene. Such a pathogenic variant is supposed to be pathogenic due to a loss of function, thus the final disease phenotype is neither aHUS nor C3G, combined with AS.

Generally, it seems that *CFB* c.2054_2057del (p.Ser687Profs^∗^16) variant associated with renal injury may be under-recognized both clinically and on biopsy. The findings in our report emphasize the importance of the extension of genetic analyses in the proband. Of course, there are some limitations in our study. Although the plasma C3 or FB levels are nearly normal, which might not deny mild-moderate continuous activity of the AP in the scenarios, detailed complement investigations (e.g., the measurement of Ba, Bb, C3a, C5a, and mac) and functional assays of mutant FB are required to clarify a precise and detailed etiological mechanism.

Alport syndrome has no radical treatment except RAS inhibitors. Considering the strong association between glomerular injury and complement activation, we speculate that anti-complement drugs combined with RAS inhibitors would be the optimal therapeutic strategy. Consequently, although the proband was treated with prednisone and Benazepril, the renal response was poor with progressive renal insufficiency. In addition, the renal injury has revealed progressive deterioration in his elder sister with a clinical course of 18 years.

## Conclusion

This rare sibling case highlights that the extension of genetic analyses in the proband is helpful for the diagnosis and understanding of some family cluster renal diseases. The study also suggests that the novel *CFB* variant has a significant pathogenic role in the process of renal injury in the presence of *COL4A5* variant and expands our understanding of FB associated glomerular nephropathy.

## Data Availability Statement

Further clinical data and images of this case are available from the corresponding author upon reasonable request.

## Ethics Statement

This study was approved by the Medicine Ethics Committee of Southeast University affiliated Zhongda Hospital. The patients/participants provided their written informed consent to participate in this study. Written informed consent was obtained from the proband and his parents for the publication of any potentially identifiable images or data included in this article.

## Author Contributions

X-lZ, Y-lW, and YT performed the medical care of the patient. BW and B-cL conceived and designed the study. F-mW drafted the manuscript. BW, F-mW, and YY critically revised the manuscript for important intellectual content. All authors read and approved the final manuscript.

## Conflict of Interest

The authors declare that the research was conducted in the absence of any commercial or financial relationships that could be construed as a potential conflict of interest.
